# Stress–Strain Index Map: A New Way to Represent Corneal Material Stiffness

**DOI:** 10.3389/fbioe.2021.640434

**Published:** 2021-03-11

**Authors:** Haixia Zhang, Ashkan Eliasy, Bernardo Lopes, Ahmed Abass, Riccardo Vinciguerra, Paolo Vinciguerra, Renato Ambrósio, Cynthia J. Roberts, Ahmed Elsheikh

**Affiliations:** ^1^School of Engineering, University of Liverpool, Liverpool, United Kingdom; ^2^School of Biomedical Engineering, Beijing Key Laboratory of Fundamental Research on Biomechanics in Clinical Application, Capital Medical University, Beijing, China; ^3^Department of Mechanical, Materials and Aerospace Engineering, School of Engineering, University of Liverpool, Liverpool, United Kingdom; ^4^Department of Production Engineering and Mechanical Design, Faculty of Engineering, Port Said University, Port Fouad, Egypt; ^5^Department of Ophthalmology, Humanitas San Pio X Hospital, Milan, Italy; ^6^The School of Engineering, University of Liverpool, Liverpool, United Kingdom; ^7^Humanitas Clinical and Research Center, IRCCS, Rozzano, Italy; ^8^Department of Biomedical Sciences, Humanitas University, Milan, Italy; ^9^Department of Ophthalmology, Federal University of São Paulo (UNIFESP), São Paulo, Brazil; ^10^Department of Ophthalmology, Federal University of the State of Rio de Janeiro (UNIRIO), Rio de Janeiro, Brazil; ^11^Department of Ophthalmology and Visual Sciences and Biomedical Engineering, The Ohio State University, Columbus, OH, United States; ^12^Beijing Advanced Innovation Centre for Biomedical Engineering, Beihang University, Beijing, China; ^13^NIHR Biomedical Research Centre for Ophthalmology, Moorfields Eye Hospital NHS Foundation Trust, UCL Institute of Ophthalmology, London, United Kingdom

**Keywords:** corneal biomechanics, keratoconus, microstructure, *in vivo*, SSI, map, stress strain index

## Abstract

**Purpose:**

To introduce a new method to map the mechanical stiffness of healthy and keratoconic corneas.

**Methods:**

Numerical modeling based on the finite element method was used to carry out inverse analysis of simulated healthy and keratoconic corneas to determine the regional variation of mechanical stiffness across the corneal surface based on established trends in collagen fibril distribution. The Stress–Strain Index (SSI), developed and validated in an earlier study and presented as a parameter that can estimate the overall stress–strain behavior of corneal tissue, was adopted in this research as a measure of corneal stiffness. The regional variation of SSI across the corneal surface was estimated using inverse analysis while referring to the common features of collagen fibrils’ distribution obtained from earlier x-ray scattering studies. Additionally, for keratoconic corneas, a method relating keratoconic cone features and cornea’s refractive power to the reduction in collagen fibril density inside the cone was implemented in the development of SSI maps. In addition to the simulated cases, the study also included two keratoconus cases, for which SSI maps were developed.

**Results:**

SSI values varied slightly across corneal surface in the simulated healthy eyes. In contrast, both simulated and clinical keratoconic corneas demonstrated substantial reductions in SSI values inside the cone. These SSI reductions depended on the extent of the disease and increased with more considerable simulated losses in fibril density in the cone area. SSI values and their regional variation showed little change with changes in IOP, corneal thickness, and curvature.

**Conclusion:**

SSI maps provide an estimation of the regional variation of biomechanical stiffness across the corneal surface. The maps could be particularly useful in keratoconic corneas, demonstrating the dependence of corneal biomechanical behavior on the tissue’s microstructure and offering a tool to fundamentally understand the mechanics of keratoconus progression in individual patients.

## Introduction

A large body of research carried out over the last two decades affirmed the important role played by corneal biomechanics in several applications including refractive surgeries (Roberts, 2002), measurement of intraocular pressure (IOP) (Böhm et al., 1997; Huseynova et al., 2014), quantifying keratoconus (KC) progression (Ortiz et al., 2007; Vinciguerra et al., 2016a), and assessing the effectiveness of cross-linking (CXL) treatment of ectasia (Kling and Hafezi, 2017). The research has repeatedly confirmed the need for a reliable method for the estimation of corneal biomechanics *in vivo*, and subsequent efforts have led to the development of methods to address this need (Ma et al., 2018). These efforts first resulted in the biomechanical parameters produced by the Ocular Response Analyzer (ORA), namely, the Corneal Resistance Factor (CRF) and the Corneal Hysteresis (CH), both of which are related to the viscoelasticity of the tissue (Shah et al., 2006, 2009; Mohammadpour et al., 2015). Still, CRF is weighted by elasticity due to its empirical development that maximized correlation to central corneal thickness (Luce, 2005; Hallahan et al., 2014). This was followed by the development of the Brillouin microscopy (BM) that assesses the tissue’s longitudinal modulus, and the Corneal Visualization Scheimpflug Technology (CorVis ST), which provided a number of Dynamic Corneal Response (DCR) parameters, including the Stiffness Parameter (SP), that correlate with corneal overall stiffness (Scarcelli et al., 2012; Vinciguerra et al., 2016b). Moreover, statistical analysis and machine learning were used to produce the indices CBI and TBI, respectively, which rely on CorVis ST and Pentacam output to detect early signs of keratoconus (Luce, 2005; Hallahan et al., 2014).

A most recent development has been the Stress–Strain Index (SSI), provided by the CorVis ST to estimate the overall stress–strain behavior of corneal tissue (Eliasy et al., 2019). With this information, the tangent modulus (Et), a measure of the tissue’s material stiffness, could be estimated at any load or stress. This point is of particular importance since biological tissues (including corneal tissue) have nonlinear stress–strain behavior, which means that Et does not have a unique value but increases with applied load and the corresponding deformation (Dupps and Wilson, 2006).

An earlier study has shown evidence of SSI’s independence of corneal thickness and IOP, and demonstrated its increase with age (Eliasy et al., 2019). This study attempts to answer an important question on the regional variation of corneal material stiffness, represented by the SSI, in both healthy and KC eyes. It sought to demonstrate how a single SSI value obtained from the CorVis ST for an eye, either healthy or KC, could be translated into a map by adding geometric information and using knowledge about collagen fibril density to show the variation of SSI across corneal surface. This map could enable visualization of the effect of the disease on the affected area, and help improve fundamental understanding of the mechanics of keratoconus progression in individual patients.

## Materials and Methods

The SSI maps rely on the microstructure of corneal tissue, and in particular the distribution of collagen fibrils (Zhou et al., 2019). Assuming that the SSI regional variation follows the distribution of collagen fibrils is based on evidence establishing collagen fibrils as the main load-carrying components of corneal tissue, and hence being responsible for corneal stiffness (Daxer et al., 1998; Bell et al., 2018). Relying on the link between microstructure and stiffness distribution was possible for five reasons; first, there is a considerable number of microstructure maps available for both healthy and keratoconic corneas (Boote et al., 2006; Meek and Boote, 2009; Niederer and McGhee, 2010; Studer et al., 2010; Pijanka et al., 2013; Ho et al., 2014; Pandolfi et al., 2019; Zhou et al., 2019; Zvietcovich et al., 2019; Li et al., 2020; Rodriguez-Garcia et al., 2020). Second, there is a strong consistency in microstructure distribution in healthy corneas with little variation between individuals. In an earlier study (Zhou et al., 2019), in which comparisons were held between the microstructure maps of six healthy corneas, the standard deviations of collagen fibril contents (in the central 6 mm-diameter area) within the 45° sectors surrounding the superior–inferior and the temporal–nasal directions were 2.8 and 2.9%, respectively. Further, the corresponding standard deviation in the content of circumferential fibrils at 11 mm diameter was limited to 1.8%. Another study observed consistency in collagen fibril diameter in the central region of five human corneas with little variation between specimens (Boote et al., 2003), and a further study reported a midline symmetry between left and right eyes when preferentially aligned fibrils were compared (Boote et al., 2006).

Third, there is evidence that the areas of keratoconic corneas outside the disease cones maintained the same microstructure features as healthy corneas (Hayes et al., 2007; Zhou et al., 2020a). In an *ex vivo* microstructure study, the areas outside the cone in keratoconic corneas were compared with corresponding areas in healthy corneas in terms of the collagen fibril distribution (Zhou et al., 2020b). The study reported no significant differences between the two groups in the contents of horizontal or vertical fibrils in the central 6-mm-diameter areas, and the same was true for the circumferential fibril content near the limbus. This particular finding is compatible with the outcome of an important study that used BM and concluded that KC was associated with a localized mechanical loss concentrated within the area of the keratoconic cone (Scarcelli et al., 2014).

Fourth, a method has recently been developed to enable identifying the transition zone between the KC cone and the surrounding tissue, in addition to the cone’s center and height, normal to the surface (Eliasy et al., 2020). In this method, an optimal sphere was fitted to the cartesian elevation map of each corneal surface within the middle 8-mm-diameter area, and the radial distances above the sphere allowed locating the cone center (as point with largest distance) and estimating its height. The height data relative to the optimal sphere was then determined along 360 equally spaced lines originating at the cone center and extending outwards using triangle-based cubic interpolation. A first derivative of the height data was calculated to determine the tangent to the surface along these lines. The second derivative was then calculated to represent the rate of change of this gradient. Since the rate of gradient change experienced a change in direction when the point of interest moves from the cone area to the surrounding healthy area, a sudden change in the sign of the rate of change in tangent gradient was indicative of an intersection with the transition zone between the pathologic area and the remaining corneal tissue. This method hence allowed estimating the transition zone (or cone boundary) and hence the cone area (Eliasy et al., 2020).

The fifth development was another method to estimate the magnitude of fibril density reduction inside the cone, κ, as a function of the cone area (A in mm^2^), the minimum corneal thickness (T in mm), and the central refractive power (P in diopters) (Zhou et al., 2020a):

(1) κ=-0.0425⁢A-0.0004⁢P+0.1⁢T+0.9727

The process of developing the SSI maps is described below in detail and the implementation was demonstrated using both simulated and clinical topography data to facilitate consideration of keratoconic cases with wide ranges of geometry, stiffness, IOP, and disease severity.

### SSI Maps for Healthy Corneas

**FIGURE 1:**
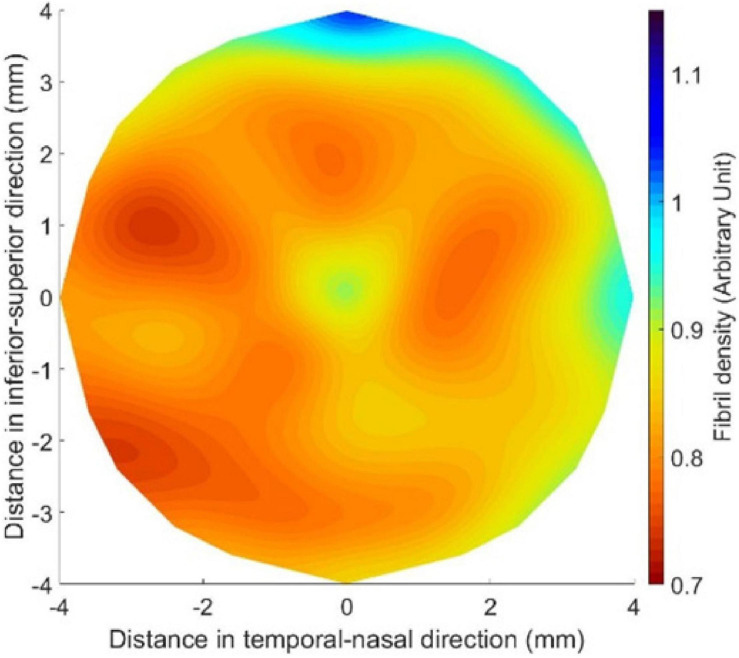
Mean fibril density in the central 8.0-mm-diameter area of healthy corneas after being fitted to Zernike polynomials of the 10th order.

Seven healthy corneas were scanned in an earlier study using x-ray scattering to determine their collagen fibril distribution (Zhou et al., 2020a). The fibril density at the measurement points, with 0.5 mm spacing in both horizontal and vertical directions, was fitted to Zernike polynomials of the 10th order, and the polynomial coefficients for the seven corneas were averaged to obtain the mean density at each point (Figure 1). The process also allowed determination of the standard deviation (SD) of fibril density at each point, and the mean value of SD (as a percentage of corresponding mean fibril density) across corneal surface was calculated in this study as 2.46 ± 0.39%. This low percentage indicated reasonable consistency of fibril distribution in healthy corneas. The direct link assumed between the fibril density distribution and the material stiffness distribution then enabled estimation of the stiffness variation from one location to another with higher fibril density leading to proportionally higher material stiffness and vice versa.

**FIGURE 2:**
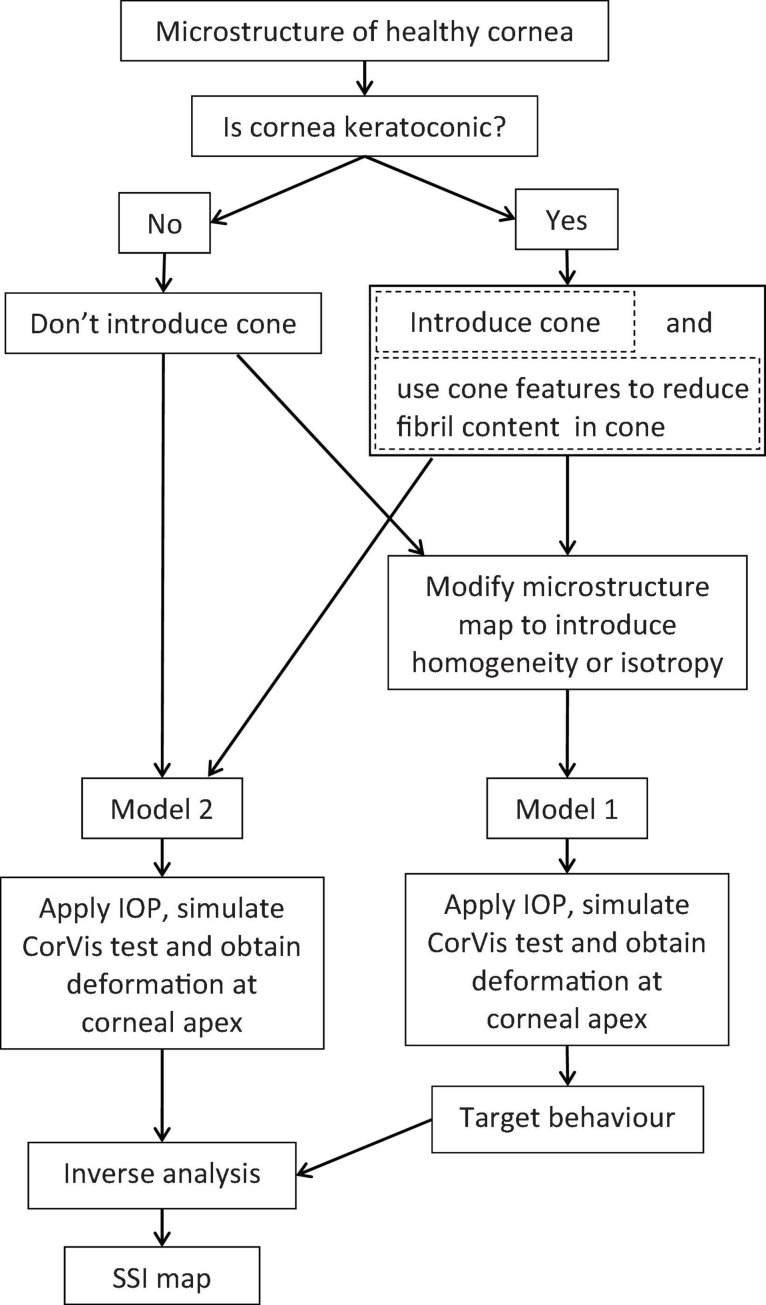
Flow chart of the process adopted to build SSI maps for healthy and keratoconic corneas.

An inverse analysis process was then established to convert the single SSI reading obtainable with the CorVis ST into an SSI map providing the value of SSI at any point across corneal surface. This process was carried out numerically and was based on an objective function that minimized the difference between the apical displacement under IOP of a cornea model (Model 1, Figure 2) adopting a homogeneous material, whose stiffness at all locations originated from the CorVis SSI value, and another cornea model (Model 2, Figure 2), for which the distribution of stiffness followed the mean distribution of fibril density for healthy corneas (Figure 1). The process started with assuming a stiffness level (i.e., a value for SSI, and hence a stress–strain behavior pattern) at each element integration point in Model 2 while observing that the ratios between these stiffness levels matched the ratios between the fibril contents at the same locations. Depending on how the resulting apical displacement of this model (Model 2) compared with the apical displacement of Model 1 (where all integration points had the same SSI), the stiffness levels adopted in Model 2 were either increased or increased by the same percentage change. These trials continued until there was a match between the apical displacements of Models 1 and 2, where this match was judged by the following objective function:

(2) $$\begin{array}{c} \mathrm{Root}\,\mathrm{mean}\,\mathrm{square}\\ \mathrm{error}\,\left(\mathrm{RMSE}\right) \end{array}=\sqrt{\frac{\sum_{i=1}^{\text{n}}{\left(\delta_{i\,\mathrm{Model1}}-\delta_{i\,\mathrm{Model2}}\right)}^{2}}{\text{n}}}$$

In this equation, δ is the apical displacement of the two models: Model 1 with a homogeneous material model and Model 2 with a stiffness distribution that matched the collagen fibril distribution; i refers to different IOP application steps and n is the total number of IOP steps.

In the analysis, a wide range of the percentage change (used with the stiffness levels in Model 2) was applied and the RMS associated with each change was determined. The percentage change values and the resulting RMS were then fitted to a third-order polynomial, and the percentage change in stiffness that corresponded to the minimum value of RMS was used to generate the SSI map.

**FIGURE 3:**
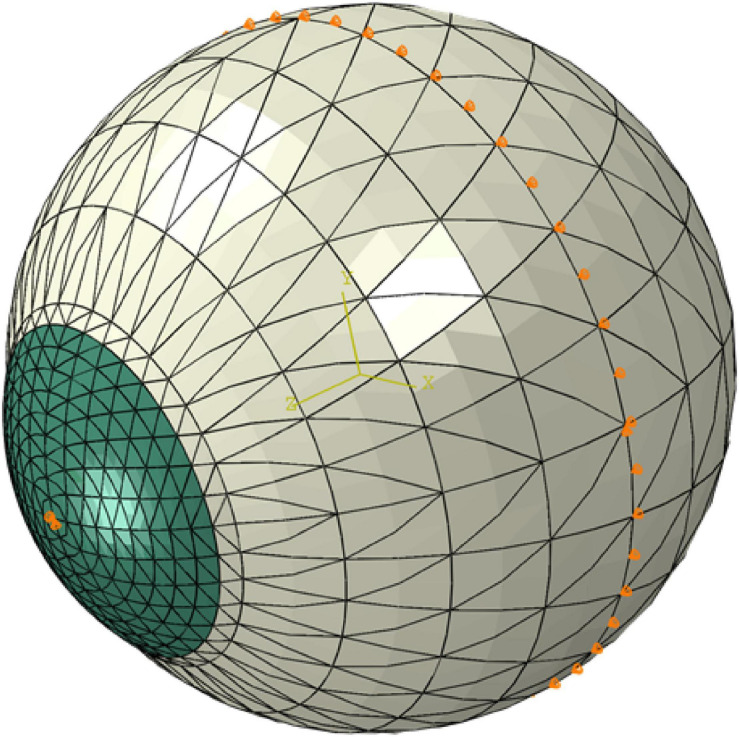
A typical finite element model showing the boundary conditions applied at the equatorial nodes and corneal apex.

The numerical models used in the study were finite element (FE) models representing the corneas and matched their assumed geometry (thickness profile, curvature, and limbal diameter). The models included 800 fifteen-noded continuum elements organized in one layer, 10 cornea element rings, and 10 sclera element rings (Figure 3). The models had a fluid cavity filled with an incompressible fluid with density 1,000 kg/m^3^ to simulate the aqueous and the vitreous. IOP was applied through controlling the pressure in this internal fluid. This technique enabled the internal eye pressure to vary from the initial IOP with the deformation experienced under the CorVis air pressure (Eliasy et al., 2019). To prevent rigid body motion, the models were restricted axially (in anterior–posterior direction) at the equatorial nodes, and in both the temporal–nasal and superior–inferior directions at the posterior pole. The FE models adopted a constitutive material model that used the collagen fibril distribution to control the regional and angular variation of stiffness across the tissue surface (Studer et al., 2010). This constitutive model was coded in a custom-built subroutine, which was integrated with the analysis process run on Abaqus FE software (version 6.14, Dassault Systemes Simulia Inc., United States).

### SSI Maps for KC Corneas

The process to develop SSI maps for KC corneas followed a similar route with two distinctive differences (Figure 2). First, the tomography maps of KC corneas were analyzed to determine the height of the cone and the transition region between the cone and outside area using a method developed in an earlier study (Eliasy et al., 2020). This method relied on height information normal to the surface above an optimal sphere to locate the cone center. It then used the second derivative of height data along equally spaced polar lines originating at the cone center to locate the cone edge. The second different step was to use the outcome of another earlier study to estimate the reduction in fibril density within the cone caused by the disease. This fibril density reduction was based on the cone height and the location of its center relative to the cornea’s apex, in addition to the max curvature of the cornea’s anterior surface (Zhou et al., 2020a). Once the fibril reduction in the cone area was estimated, the modeling exercise described above was adopted with no change.

In order to demonstrate the construction and illustrate the benefits of the SSI maps, a parametric study was carried out to cover cases with a KC cone of radius of 0.5, 1.0, 1.5, and 2.0 mm; a distance between cone center and corneal apex of 0.0, 1.0, 2.0, and 3.0 mm; and an angle of 0°, 45°, and 90° between the horizontal center line of cornea and line from apex to cone center. In all cases, the fibril density within the cone area was reduced by a maximum of 60% in steps of 10%. This maximum reduction was compatible with cases of severe KC as was found in our earlier study (Zhou et al., 2020a). Additionally, the study considered a healthy cornea with no cone.

In all cases, IOP was assumed to be 15 mmHg, with an anterior central radius of 7.8 mm, an anterior shape factor of 0.82 (shape factor = 1 - asphericity), a central corneal thickness of 545 microns, and a limbal diameter (or white-to-white distance) of 11.85 mm. These values represented the means of clinical measurements reported in the literature (Gilani et al., 2013). For simplicity, all models assumed that corneal boundary was circular. The KC cones were also all assumed to have a circular base. Most models further assumed an SSI measurement of 0.7, which represented a material stiffness level that is within both the healthy and KC ranges.

Further, in order to recognize the fact that KC leads to not only a reduction in fibril density within the cone but also a change in fibril arrangement (which has not been quantified), part of the study was repeated to consider the effect of assuming either total isotropy or random distribution at each measurement point within the KC cone.

#### Application to Clinical Data

The methods described above to generate SSI maps were implemented in two clinical cases with mild and advanced keratoconus, respectively. Tomography and biomechanical data on these two cases were obtained from the Vincieye Clinic in Milan, Italy. Institutional review board (IRB) ruled that approval was not obligatory for this record review study. However, the ethical standards set in the Declaration of Helsinki were observed. Both patients provided informed consent before using their data in research. They had a complete ophthalmic examination, including CorVis ST and Pentacam (Oculus Optikgeräte GmbH; Wetzlar, Germany) exams. Both patients had bilateral keratoconus without any previous ocular surgeries, such as collagen CXL or intracorneal rings.

## Results

### SSI Maps for Healthy Corneas

**FIGURE 4:**
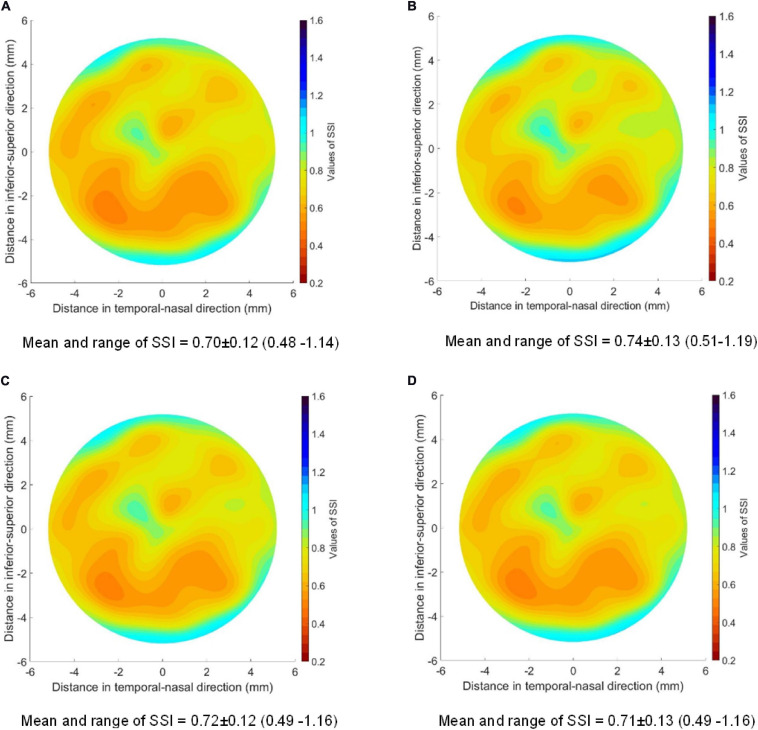
SSI maps for four healthy corneas with the same SSI of 0.7 and different geometrical features and IOP. **(A)** IOP = 15 mmHg; CCT = 545 μm; *R* = 7.8 mm; **(B)** IOP = 30 mmHg; CCT = 545 μm; *R* = 7.8 mm; **(C)** IOP = 15 mmHg; CCT = 445 μm; *R* = 7.8 mm; **(D)** IOP = 15 mmHg; CCT = 545 μm; *R* = 8.4 mm. The values of SSI for each case were mean value ± standard deviation (range from minimum to maximum value). The peripheral thickness was set to 150 microns more than CCT in these models.

Figure 4A shows the SSI map for an idealized healthy cornea with an IOP of 15 mmHg, an SSI of 0.7, and the geometric features described above. Figure 4 also includes cases in which (B) IOP was increased to 30 mmHg, (C) central corneal thickness (CCT) reduced from 545 μm to 445 μm, and (D) anterior central radius of curvature (R) changed from 7.8 to 8.4 mm. In all cases, the SSI distributions followed the fibril density distribution for healthy corneas depicted in Figure 1 with almost complete independence of corneal geometry and IOP.

### SSI Maps for KC Corneas

**FIGURE 5:**
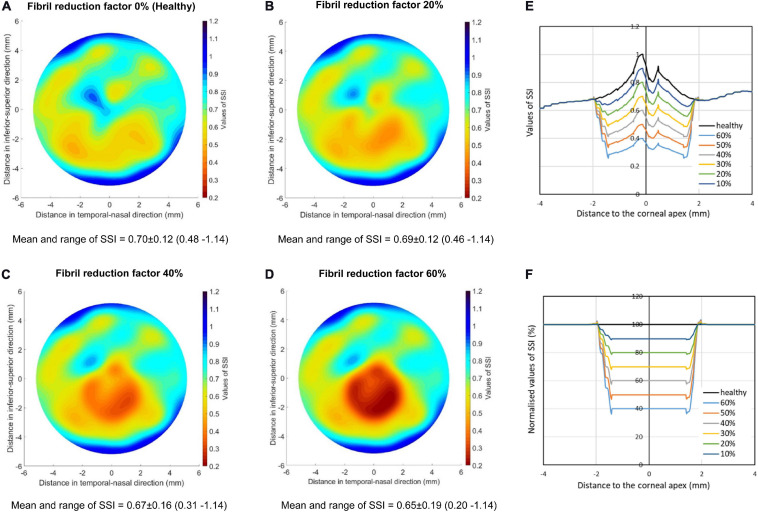
SSI maps for KC corneas with cones experiencing fibril reductions of 0 **(A)**, 20% **(B)**, 40% **(C)**, and 60% **(D)**. The cones had 2.0 mm radius and were located on the vertical central meridian of the cornea. In all cases, the cones’ centers were at 1.0 mm away from corneal apex. The values of SSI provided for each case represented the mean ± standard deviation and range from minimum to maximum value. **(E,F)** depict the SSI values along the horizontal central meridian of the cornea before **(E)** and after **(F)** normalization relative to the corresponding SSI values of the healthy cornea.

The first parameter to consider is the fibril density reduction within the cone area, which varied from 0 (in healthy corneas) to 60% (in severe KC cases). The results presented in Figure 5 are for corneas with 2.0-mm-radius cones, whose centers were at 1.0 mm away from corneal apex and located on the vertical central meridian. The figure shows the SSI maps for cones with fibril reduction factors of 0, 20, 40, and 60%. It also shows the SSI values along the horizontal central meridian of the cornea before and after normalization relative to the corresponding SSI values of the healthy cornea.

In these analyses, the SSI of the healthy cornea was assumed at 0.7, while for KC corneas, an optimization process was carried out to determine the value of the overall SSI (the CorVis ST single SSI reading), for which the SSI values in the parts outside the cones of the KC corneas would remain at the same level as in the corresponding areas in the healthy eye. This exercise was carried out for fibril density reduction factors of 20, 40, and 60%.

Figure 5 shows that with the successive reductions in local fibril density, the SSI reduced in the cone area. There was also a gradual reduction in the overall SSI from 0.7 in the healthy cornea to 0.597, 0.469, and 0.298 with fibril reductions in the cone of 20, 40, and 60%, respectively.

Comparisons were also held to show the effect of cone fibril arrangement on the results. The comparisons were between three KC models, all with the same fibril density reduction (60%) in the cone area. While one model maintained the same fibril arrangement as in healthy tissue, another assumed uniform and isotropic fibril distribution. A third model was also included in which the variation of fibril density from point to point within the cone, as well as the anisotropy at each point, was made random (using the Random function in Microsoft Excel, Version 2020). However, in all three models, it was ensured that the total fibril content within the cones remained the same.

**FIGURE 6:**
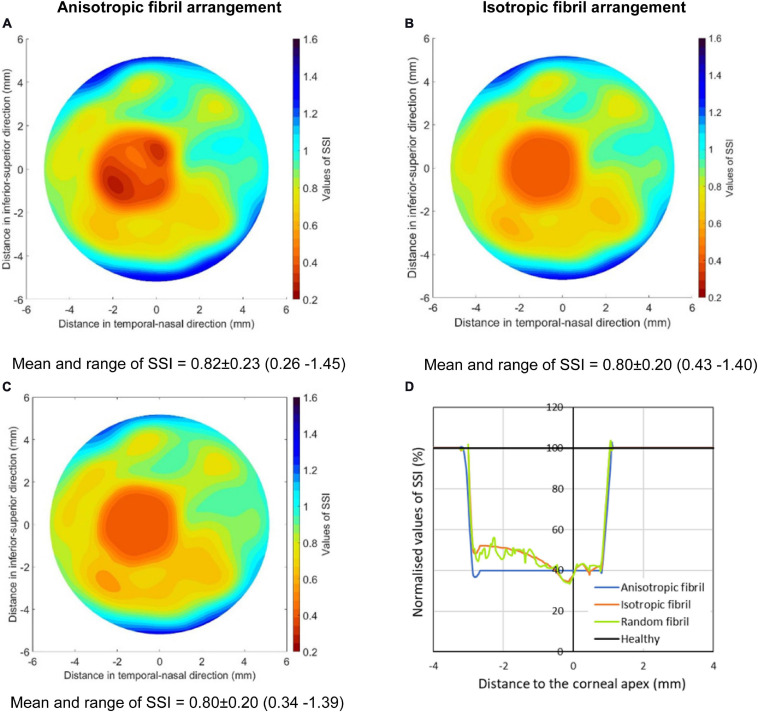
SSI maps of three KC cases including **(A)** a model with the same fibril arrangement as in healthy tissue but with 60% fibril reduction in the cone, **(B)** a model with uniform and isotropic fibril distribution in the cone, and **(C)** a model in which the variation of fibril density from point to point within the cone, as well as the anisotropy at each point, was made random. All three models had the same total fibril content within the cones. **(D)** presents normalized SSI values along the horizontal central meridian of the cornea for all three models.

**FIGURE 7:**
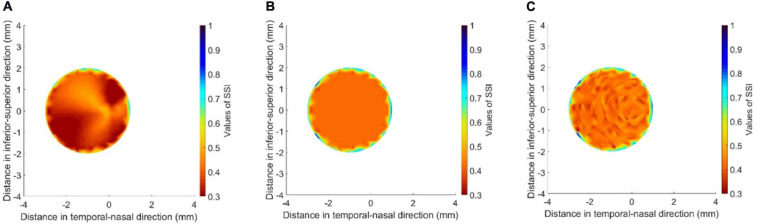
SSI maps for the cone areas in the three models depicted in Figure 6 including **(A)** a model with the same fibril arrangement as in healthy tissue but with 60% fibril reduction in the cone, **(B)** a model with uniform and isotropic fibril distribution in the cone, and **(C)** a model in which the variation of fibril density from point to point within the cone, as well as the anisotropy at each point, was made random.

All three models had a 2.0-mm-radius cone, whose center was 1.0 mm away from apex and located on the horizontal central meridian. The results presented in Figures 6, 7 show large effects of changing the fibril arrangement on the SSI values inside the cones with mean variations in SSI of 15.7 and 15.5% in the second and third models, respectively, relative to the first model. However, outside the cones, there was very little change in SSI values or their distribution, which led to the mean variations in SSI across corneal surface to remain below 2%. This result, and the fact that the change in fibril arrangement with KC progression has not been quantified (Zhou et al., 2020a), allowed the rest of the study to assume a reduction in fibril density within the cone but with no corresponding change in fibril arrangement.

**FIGURE 8:**
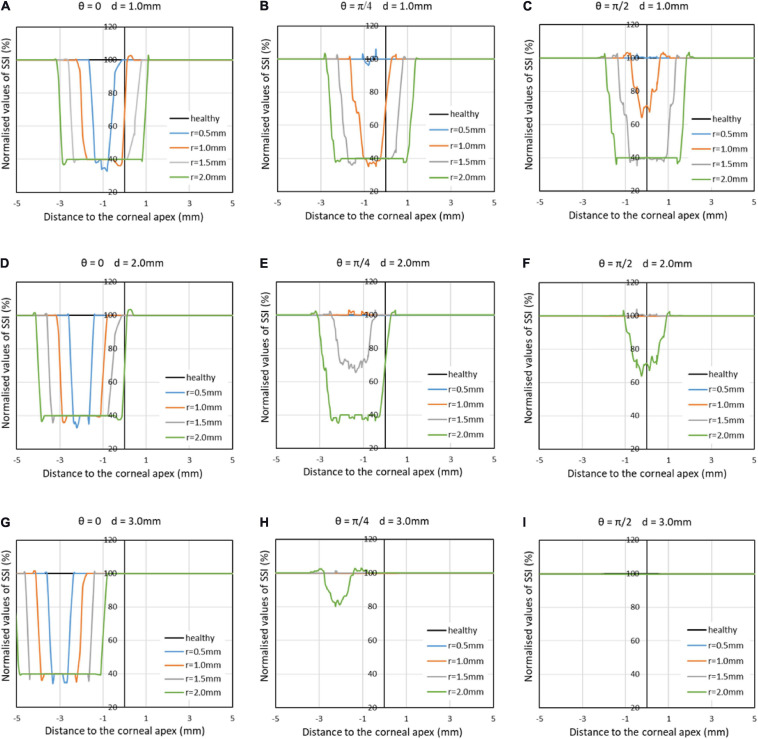
Normalized SSI values along the horizontal central meridian of the cornea for several cases, in which the KC cone had a radius (r) of 0.5, 1.0, 1.5, or 2.0 mm; a distance between cone center and corneal apex (d) of 1.0, 2.0, or 3.0 mm; and an angle (θ) of 0°, 45°, or 90° between the horizontal center line of cornea and line from apex to cone center. The max fibril density reduction (60%) was considered in all cases.

The next step was to consider the effect of cone size, distance from cone to apex, and location of cone center on the SSI maps. Here, the focus was on SSI values along the horizontal central meridian of the cornea – normalized against the corresponding values obtained for healthy corneas (Figure 8). In all cases presented in this figure, the max fibril density reduction (60%) was considered. The results show a gradual expansion in the area affected by fibril density reduction with growth in cone size. With an emphasis in the figure on the SSI distribution along the horizontal meridian, changes in the distance of cone center away from apex led to shifting the effect in cases where the cone center was on the horizontal meridian (theta = 0°) or reducing the effect when the cone center was on the vertical meridian (theta = 90°). In cases where the cone center was on a diagonal meridian (theta = 45°), the result of increasing the distance away from the apex was a combination of shifting the reduction in SSI values away from the center in addition to an increase in the magnitude of SSI reduction.

#### Application to Clinical Data

**FIGURE 9:**
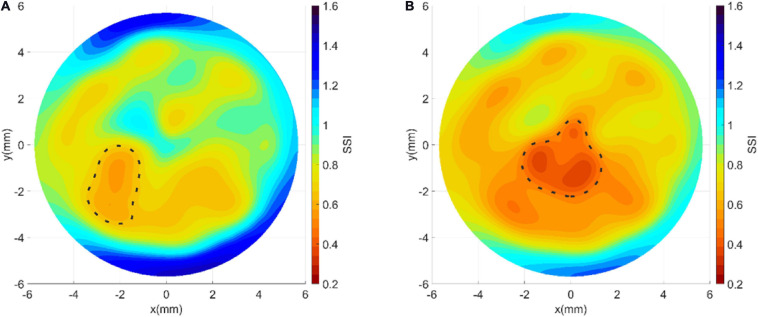
Stress–strain maps obtained for **(A)** a mild and **(B)** an advanced keratoconic cornea demonstrating the stiffness reduction in the cone area.

In the mild keratoconic case, the patient age was 21 years, cornea’s min thickness was 454 microns, refractive power was 46.1 D, and SSI was 0.86. Using the study of cone features published earlier (Eliasy et al., 2020), the cone mean radius was estimated at 1.37 mm, cone height was 0.017 mm, and cone area was 4.523 mm^2^. Also, using Equation 1, the collagen fibril reduction factor (within the cone area) was 19%. On the other hand, the advanced keratoconic case had an age of 27 years, a min thickness of 377 microns, a refractive power of 65 D, and an SSI value of 0.43. Further, cone mean radius was estimated at 1.57 mm, cone height was 0.258 mm, cone area was 6.562 mm^2^, and fibril reduction factor was 41%. The two cases were analyzed to generate the SSI maps following the process explained above (Figure 2), and the results are depicted in Figure 9. The maps show the localized softening within the cone areas, which was particularly evident in the case with advanced keratoconus.

## Discussion

The recent development of the SSI (Eliasy et al., 2019), as a measurement of corneal material stiffness, promised a strong potential to customize processes that interact or interfere mechanically with the cornea including the planning of refractive surgery for normal patients, as well as the selection of corneal implants, the measurement of IOP, and assessing the effectiveness of CXL treatment in keratoconus. The subsequent quantification of the regional variation of SSI in this study and the development of SSI maps offer further opportunities in offering a tool to improve fundamental understanding of the mechanics of keratoconus progression in individual patients. As the SSI maps provide a direct method to quantify the stiffness deterioration within the cone area and how this deterioration worsens with progression or reduces with treatment, they can lead to better understanding of the effect of the disease and more informed management.

Based on earlier evidence recognizing the collagen fibrils as the main load-carrying components of the cornea, the study relied on the microstructure maps available for healthy corneas and detailing the fibril density and orientation across corneal surface (Daxer et al., 1998; Bell et al., 2018). The availability of this information and the consistency of microstructure details among healthy corneas provided a suitable route to the development of SSI maps in this study.

With KC corneas, there had been further challenges related to the ability to locate the cone transition to an area of structural difference and estimate the change in fibril density both within and outside the cone area. Microstructure data obtained for KC corneas and reported earlier indicated that the areas outside the cones were not significantly affected by the disease (Scarcelli et al., 2014; Zhou et al., 2020a). Other studies provided a much-needed method for the determination of the cone location and geometric features, including its area, center, and height based on tomography data (Eliasy et al., 2020). A further study allowed linking these features to the fibril density reduction inside the cone (Zhou et al., 2020a). Together, these developments enabled the estimation of the fibril density distribution that considered the effect of KC on density reduction.

Before deriving SSI maps, an evaluation is required to know whether the cornea is healthy or keratoconic. If the cornea is healthy, the analysis to produce the stiffness map will proceed based on tomography data and the SSI CorVis ST measurement. If, on the other hand, the cornea is keratoconic, the cone features are used to estimate the fibril reduction within the cone area, and with this information, the analysis to produce the stiffness maps can start.

The cornea assumes a shape (topography) that satisfies the mechanical equilibrium between the applied pressure (IOP) and corneal stiffness; this corneal stiffness has two components: the material stiffness and the geometric stiffness. Therefore, knowing the IOP and both the material stiffness and the geometric stiffness would allow estimating corneal topography. However, for a 3D object like the cornea with a significant thickness variation across the surface, it would be difficult to estimate the material stiffness distribution from the topography maps, as the topography is not solely dependent on the material stiffness. For this reason, we sought to determine the material stiffness maps from first principles – based on reliance on the tissue microstructure. Once these maps are developed, weak areas can be identified to show the scale of stiffness deterioration associated with KC progression.

The reliance on the microstructure to control the stiffness variation from one point to another across the cornea provided a simple route to the development of stiffness maps but had its drawbacks. The assumption that all healthy corneas (and the areas outside cones in KC corneas) have the same microstructure was necessary for our study and will perhaps remain necessary until a technique for the *in vivo* mapping of the microstructure is developed. However, this assumption meant that the small variations that exist between individual eyes were not considered. Another limitation of the study is the reliance on a method developed earlier to relate cone geometric features to the fibril density reductions within the cone (Zhou et al., 2020a). That study relied on the analysis of only seven KC corneas, which were all that was available to the researchers. Therefore, it will be necessary in the future to add more KC scans to improve the resulting estimation of fibril density reduction and disrupted orientation.

The goal of this study in producing stiffness maps of the cornea is shared with other technologies, including most notably BM. BM relies on an inelastic scattering process, which is associated with a medium’s acoustic phonons or density fluctuations. Measurement of spectral changes provides information on the phonons’ properties and is highly correlated with viscoelastic properties (Scarcelli and Yun, 2008). While it was unable to estimate the stress–strain behavior of tissue, the technique was successful in estimating the tissue’s longitudinal modulus and how it varies in 3D across corneal surface and through the tissue’s thickness. The technique was applied to patients who underwent corneal CXL and demonstrated a significant increase in stiffness after the procedure (Scarcelli et al., 2013). Nonetheless, the longitudinal modulus and the tangent modulus are two independent elastic properties, and their individual influences on evaluating overall clinical biomechanical response are not yet well linked (Yun and Chernyak, 2018; Eliasy et al., 2019).

This study provided a method to map the distribution of mechanical stiffness of the cornea, which can be used *in vivo* and in both healthy and keratoconic eyes. The resulting maps can potentially offer a tool to improve fundamental understanding of the mechanics of keratoconus progression in individual patients.

## Data Availability Statement

The data analyzed in this study is subject to the following licenses/restrictions: There is a material transfer agreement in place. Requests to access these datasets should be directed to AsE, ashkan@eliasy.com.

## Ethics Statement

The studies involving human participants were reviewed and approved by the Vincieye Clinic in Milan, Italy and University of Liverpool in Liverpool, United Kingdom. The patients/participants provided their written informed consent to participate in this study.

## Author Contributions

HZ and AsE drafted the manuscript, carried out the study, performed the analysis, and acquired the results. AsE developed the software used in the work and supervised the conduct of the study. BL and AA interpretated the data and supported the analysis. RV, PV, RA, and CR critically reviewed the manuscript, interpreted the data, and contributed in the design of the work. AhE designed and conceptualized the study, supervised the entire project, drafted the manuscript, and interpreted the data. All authors have reviewed and approved the submitted version, and agreed both to be personally accountable for the author’s own contributions and to ensure that questions related to the accuracy or integrity of any part of the work, even the ones in which the author was not personally involved, are appropriately investigated and resolved, and the resolution is documented in the literature.

## Conflict of Interest

RV, PV, RA, CR, and AhE are consultants for Oculus. The remaining authors declare that the research was conducted in the absence of any commercial or financial relationships that could be construed as a potential conflict of interest.
